# Assessing the Potential of Olive Stone Powder as a Bitumen Biopolymer Through Physical, Chemical, and Rheological Characterization

**DOI:** 10.3390/polym18050661

**Published:** 2026-03-08

**Authors:** Ozgur Ozcan, Halil Ibrahim Yumrutas, Abdulgazi Gedik, Sedat Ozcanan, Mustafa Yurdabal Apak

**Affiliations:** 1 Department of Civil Engineering, Faculty of Engineering, Sirnak University, 73000 Sirnak, Türkiye; 2 Department of Civil Engineering, Faculty of Engineering and Natural Science, Karabuk University, 78050 Karabuk, Türkiye; 3 Department of Civil Engineering, Faculty of Engineering and Natural Sciences, Malatya Turgut Ozal University, 44900 Malatya, Türkiye; 4 Faculty of Civil Engineering, Istanbul Technical University, 34469 Istanbul, Türkiye; 5 Department of Civil Engineering, Faculty of Engineering and Architecture, Istanbul Gelisim University, 34310 Istanbul, Türkiye

**Keywords:** olive stone powder, biopolymer, asphalt binder, sustainable pavement materials, recycling, rheological performance

## Abstract

The present study aims to investigate the feasibility of utilizing olive stone powder (OSP), an agricultural by-product, as a modifier for bituminous binders. OSP was incorporated into a neat bitumen at dosages of 2%, 4%, 6%, and 8% by weight, and the modified binders were subjected to comprehensive laboratory tests along with the unmodified reference binder. The evaluation framework included physical, rheological, and chemical characterization tests. The results of physical tests indicate that, although the addition of OSP led to a slight increase in binder stiffness, it effectively reduced temperature susceptibility while maintaining workability within acceptable limits. Rheological results showed that OSP modification improved rutting resistance at high temperatures, while low-temperature performance was preserved at 2% and 4% OSP contents; however, increased stiffness at higher dosages (6% and 8%) may increase thermal cracking susceptibility. Chemical analyses confirmed that OSP was homogeneously dispersed within the bitumen matrix and improved binder behavior primarily through physical interactions, while also enhancing thermal stability. Overall, the results indicate that OSP behaves as a biopolymer-based, filler-like modifier and provides performance improvements primarily through physical structuring. With these characteristics, OSP offers an environmentally friendly and economical solution for bituminous binders and represents a promising option for sustainable pavement materials.

## 1. Introduction

Asphalt pavements constitute the most used pavement type in highway engineering applications. [1,2]. It is estimated that more than 25 million kilometers of roadways worldwide will be paved with asphalt pavements over the next 30 years [3]. Global annual bitumen consumption is estimated to be approximately 120 million tons, of which nearly 85% is utilized in road construction and pavement applications [4,5]. The continuous increase in heavy vehicle traffic, inadequate maintenance practices, and changing climatic conditions have progressively raised performance demands for asphalt pavements. In this context, the inherent temperature susceptibility of bitumen, its tendency toward fatigue under repeated loading, and its vulnerability to oxidative aging significantly contribute to the development of permanent deformation (rutting) and various cracking modes. Consequently, extensive research efforts have focused on improving asphalt pavement performance through bitumen modification using a wide range of additives [6,7].

Bitumen modifiers are commonly categorized as polymeric and non-polymeric additives. Polymer-modified binders, particularly those incorporating styrene–butadiene–styrene (SBS) and ethylene–vinyl acetate (EVA), are widely used due to their effectiveness in improving rutting and cracking resistance [8,9]. However, the high cost and environmental footprint of these synthetic polymers have motivated the search for more sustainable alternatives. In this context, increasing attention has been directed toward bio-based and waste-derived modifiers, including biopolymers, which offer the potential to enhance binder performance while reducing environmental impact and material costs. In recent years, research interest in the utilization of organic wastes for bitumen modification has increased markedly. Organic waste is readily available and renewable raw materials, as it is generated as by-products of various industrial and agricultural processes [10]. It is estimated that global annual production of organic waste exceeds 100 billion tons, accounting for a substantial portion of total waste generation worldwide [11,12]. Developing new application areas for organic waste, which possess significant value-added potential when incorporated into engineering products, can contribute not only to reducing environmental pollution but also to improving resource efficiency and promoting sustainability [13]. Owing to their phenolic compounds, fatty acids, and lignocellulosic constituents, organic waste-derived materials have been reported to retard the oxidative aging of bitumen and enhance high-temperature performance, thereby mitigating permanent deformation (rutting) and cracking phenomena. Materials such as wood sawdust [14], lignin [15], and waste cooking oils [16] or bio-based oils [17] represent some of the organic wastes investigated for bitumen modification. Within this framework, olive stone (OS), a widely generated by-product of the olive oil processing industry, has emerged as a promising biopolymer candidate due to its global abundance and potential antioxidant content. OS is separated from wet olive pomace generated during olive oil production and is handled differently from dried pomace residues [18,19]. OS accounts for approximately 10–30% of the olive fruit by weight [20], while only about 15–20% of the fresh olive mass is converted into olive oil [21]. The olive oil market has experienced rapid growth in recent decades, with global production increasing from 1.5 million tons in the 1990/1991 season to approximately 3.4 million tons in the 2024/2025 season. Moreover, demand for olive oil continues to rise steadily worldwide [22,23]. As a lignocellulosic material composed primarily of cellulose, hemicellulose, and lignin, OS contains significant amounts of phenolic compounds such as oleuropein, flavonoids, lignin, and phenolic acids, which are known for their antioxidant properties [24,25]. These compounds remain largely stable up to temperatures of approximately 180 °C when OS is used in its raw and dry form, with thermal degradation occurring predominantly between 180 and 500 °C [26].

Several studies have examined the use of by-products from olive oil production in bitumen and asphalt materials, mainly focusing on olive pomace and ash-based olive waste materials, as summarized in Table 1.

Despite the growing body of research on olive-derived and other bio-based modifiers for bituminous binders, as summarized in Table 1, existing studies have predominantly focused on incinerated olive residues or olive waste ash, where the combustion process removes the organic and lignocellulosic constituents and results in materials that primarily behave as inorganic, mineral-type fillers. While such ash-based additives have demonstrated certain performance benefits, their interaction mechanisms with bitumen are fundamentally different from those of raw biomass due to the loss of polymeric structure, functional groups, and naturally occurring antioxidant components during incineration [27,28,36]. Moreover, the incineration process itself is associated with additional energy consumption, high-temperature processing, and potential environmental impacts, which contradict the sustainability objectives often attributed to bio-based modifiers. In contrast, directly ground, dry olive stone powder (OSP) retains its rich organic and lignocellulosic composition, including cellulose, hemicellulose, and lignin, as well as inherent antioxidant compounds. This enables a biopolymer-based, filler-like physical structuring mechanism within the binder rather than purely mineral stiffening, while avoiding the environmental and energetic burdens associated with combustion-based processing. Nevertheless, a systematic and integrated evaluation of raw, non-incinerated OSP as a binder modifier remains lacking in the literature, particularly with respect to jointly correlating its physical and rheological behavior with microstructural and chemical evidence. The present study is designed to address this gap.

By explicitly distinguishing raw OSP from incinerated olive residues, this study aims to clarify the role of retained organic constituents in governing binder-level, temperature-dependent performance, particularly the balance between high-temperature stiffness and low-temperature flexibility. By utilizing OS as a low-cost and renewable agricultural by-product, this research offers an environmentally sustainable alternative to conventional polymer modifiers. Accordingly, binders containing different OSP contents were evaluated through a comprehensive laboratory program focusing on temperature susceptibility, binder-level rheological indicators, and fundamental physical properties, providing an integrated assessment of the performance potential and limitations of OSP-modified binders.

## 2. Materials and Methods

### 2.1. Material

The bitumen used in this study was a B 50/70 penetration grade binder, supplied by the Batman refinery of the Turkish Oil Refineries Corporation (TÜPRAŞ), Türkiye. Prior to modification, the binder was subjected to preliminary laboratory tests to verify its compliance with the requirements of EN 12591 [42]. The characteristic properties of the base bitumen, determined through standard tests, are presented in Table 2.

**Figure 1:**
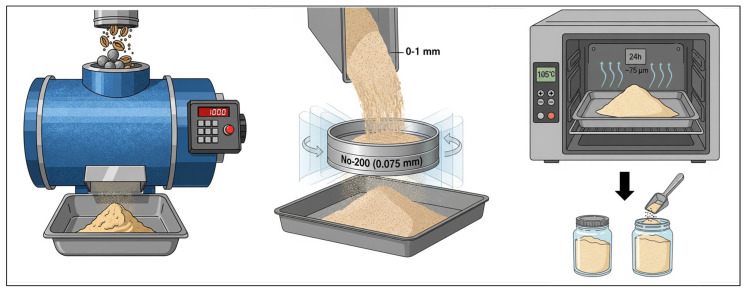
Processes for preparing OSP: milling, sieving, drying and storing.

Dry OS was obtained from the Balikesir/Edremit region and mechanically ground using a Los Angeles abrasion machine with steel balls until a particle size range of approximately 0–1 mm was achieved. The ground material was subsequently sieved through a No. 200 sieve (0.075 mm) to obtain OSP. Before the modification process, the OSP was oven-dried at 105 °C for 24 h in a wide tray and then stored in sealed glass containers. This drying procedure was applied to eliminate the adverse effects of moisture on the chemical structure and phenolic compounds of OSP [26]. The preparation procedure of OSP is illustrated in Figure 1.

**Figure 2:**
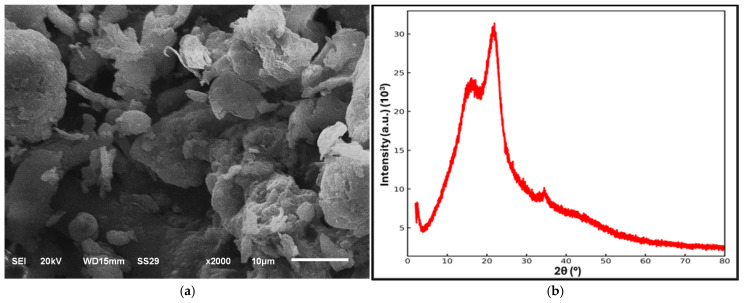
Chemical Characterization of OSP: (**a**) SEM image; (**b**) XRD spectrum.

The surface morphology, elemental composition, and crystallographic structure of OSP were investigated using scanning electron microscopy (SEM), energy-dispersive X-ray spectroscopy (EDX), and X-ray diffraction (XRD) analyses, respectively. The SEM micrograph presented in Figure 2a shows that OSP consists of irregularly shaped, heterogeneous particles with rough surface textures, which is characteristic of natural lignocellulosic materials. The XRD pattern shown in Figure 2b indicates that OSP predominantly exhibits an amorphous structure, as evidenced by the broad and low-intensity diffraction band. Weak and broad diffraction features observed around 2θ ≈ 20–22° correspond to the characteristic crystalline regions of cellulose, suggesting the presence of a limited amount of crystalline cellulose within an otherwise amorphous lignocellulosic matrix.

The elemental composition obtained from EDX analysis (Table 3) reveals that OSP is primarily composed of carbon (≈58.5%) and oxygen (≈34.8%), confirming its organic and lignocellulosic nature [43,44]. Minor amounts of inorganic elements such as Si, K, Ca, Fe, and Zn were also detected, which can be attributed to inherent mineral constituents naturally present in biomass materials.

**Figure 3:**
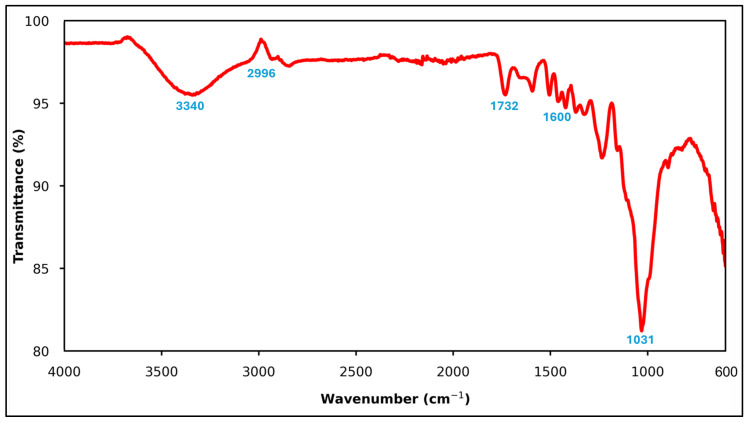
FTIR spectrum of OSP. The red line shows the FTIR transmittance spectrum, and the blue numbers denote the main characteristic absorption peaks (cm^−1^).

The FTIR spectrum of OSP confirms its lignocellulosic structure, consisting mainly of cellulose, hemicellulose, and lignin. As shown in Figure 3, the broad band around 3400 cm^−1^ corresponds to –OH stretching vibrations, indicating hydroxyl-rich polysaccharide and phenolic groups typical of plant-based biomass. The absorption near 2920 cm^−1^ is attributed to aliphatic C–H stretching, associated with organic backbone structures. A distinct peak at approximately 1730 cm^−1^ represents C=O stretching vibrations of ester and carboxylic groups, mainly related to hemicellulose and residual lipid components. The band around 1600 cm^−1^ is characteristic of aromatic C=C stretching, confirming the presence of lignin, while the strong absorption near 1030 cm^−1^ is assigned to C–O stretching of cellulose and hemicellulose. Overall, the FTIR results indicate that OSP is composed of oxygen-rich lignocellulosic constituents without evidence of synthetic polymeric functional groups, supporting its classification as a bio-based organic filler rather than a true polymer modifier.

### 2.2. Method

**Figure 4:**
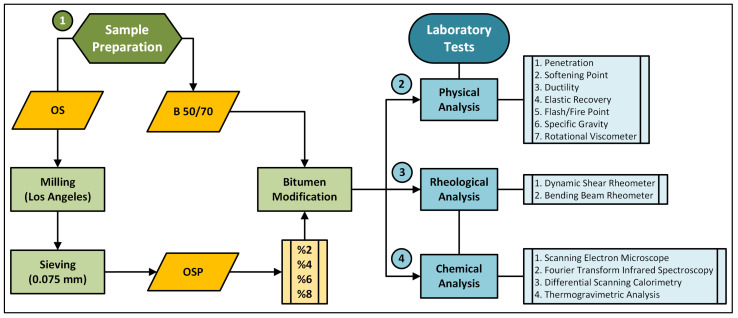
Flowchart of methodology. Green boxes represent sample preparation processes, yellow boxes denote materials, and blue boxes indicate laboratory tests.

The laboratory investigations were conducted in four sequential stages: (1) sample preparation and bitumen modification with OSP, (2) physical testing, (3) rheological testing, and (4) chemical characterization. A schematic flowchart illustrating experimental procedures is presented in Figure 4.

#### 2.2.1. Bitumen Modification with OSP

**Figure 5:**
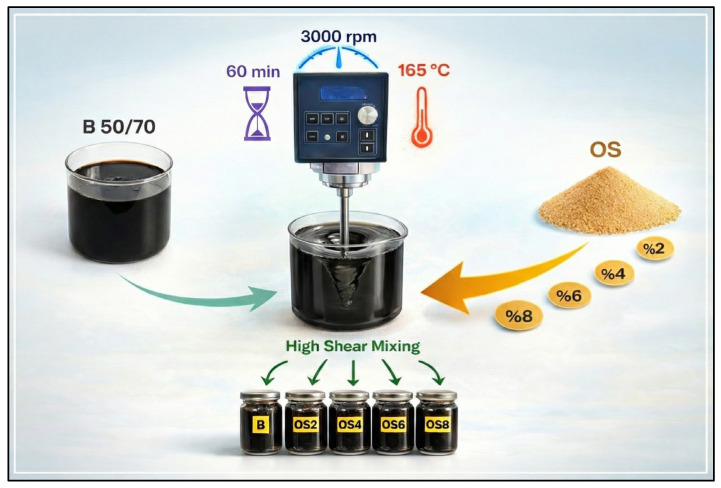
Bitumen modification protocol.

Prior to modification, the neat bitumen was conditioned at 165 °C for approximately 30 min to ensure complete fluidity. Subsequently, OSP was incorporated into 1000 g of B50/70 bitumen at dosages of 2%, 4%, 6%, and 8% by weight. The selected OSP dosage range was determined by considering the typical effective content levels of widely used polymer modifiers such as SBS and EVA, which are commonly applied within the range of approximately 3–5%. In addition, higher modifier contents are known to induce practical limitations, including excessive viscosity increase, reduced workability, and an increased risk of phase separation during storage and handling. Therefore, the selected dosage range, consistent with values reported in the literature, was intended to evaluate both the benefits and practical limitations associated with increasing OSP content [37,38,41]. The modification process was carried out by mixing the binders at 165 °C for 60 min using a high-speed mixer (Silverson Model L5M) operating at a rotational speed of 3000 rpm. The selected mixing temperature was chosen to ensure sufficiently low binder viscosity for effective dispersion of OSP while avoiding excessive oxidative aging, which is consistent with commonly adopted temperatures for polymer- and bio-based binder modification. The mixing duration of 60 min was selected based on previous studies indicating that extended mixing times are required to achieve stable and homogeneous dispersion of solid bio-derived modifiers, while limiting unnecessary thermal aging. High-shear mixing at 3000 rpm was applied to promote uniform distribution of OSP particles within the binder and to enable reliable microstructural and chemical characterization. It is well established that insufficient shear rates may hinder the identification of modifier phases and lead to misleading interpretations in chemical and morphological analyses [45]. To ensure that all samples experienced identical thermal and shear histories and to isolate the effect of OSP addition, the unmodified B50/70 control binder was subjected to the same thermal and shear conditioning, without the addition of any modifier. Following the modification process, a smear test was performed for each binder as a qualitative pre-screening method to assess homogeneity. The test involved spreading a thin film of the freshly prepared binder onto a flat surface using a spatula and visually inspecting it for signs of particle agglomeration or non-uniform distribution. All modified binders exhibited a uniform and continuous film, with no visible evidence of phase separation. The prepared samples were allowed to cool to ambient temperature and subsequently stored in a cool, dark, and airtight environment until further laboratory testing. A schematic illustration of the modification procedure is presented in Figure 5.

To prepare the modified binders, the B50/70 base binder was separately blended with OSP at four different dosages (2%, 4%, 6%, and 8% by weight). Accordingly, a total of four OSP-modified binders were produced in this study. Each binder was coded using a combination of letters and numbers, where B denotes the unmodified B50/70 binder, OS represents OSP–modified binders, and the numbers 2, 4, 6, and 8 indicate the corresponding OSP content by weight. For instance, OS2 refers to the binder modified with 2% OSP, whereas B denotes the unmodified control binder.

#### 2.2.2. Physical Analysis

First, penetration tests were conducted on all binder samples at 25 °C in accordance with ASTM D5 (2020) to determine binder consistency. Penetration values were measured by allowing a standard needle loaded with 100 g to penetrate the binder for 5 s [46]. To determine the transition temperature of bitumen from a semi-solid to a liquid state, softening point test was performed using the ring-and-ball method in accordance with ASTM D36 (2020). The softening point was determined by recording the temperature at which the steel balls dropped, with a heating rate of 5 °C/min [47]. Ductility tests were carried out following ASTM D113 (2017) to evaluate the ability of the binder to undergo permanent deformation under tensile loading. Ductility values were obtained by recording the elongation at break at 25 °C using a pulling rate of 50 mm/min [48]. The elastic recovery of the binders was evaluated at 25 °C in accordance with ASTM D6084 (2021). In this test, specimens were elongated to 200 mm, cut using scissors, and the recovered length was measured to calculate the elastic recovery percentage [49]. The specific gravity of the binders relative to distilled water was determined at 25 °C using a pycnometer in accordance with ASTM D1480 (2021) [50]. Rotational viscosity measurements were performed using a Brookfield rotational viscometer at temperatures of 135 °C and 165 °C, with a spindle rotating at 20 rpm, following ASTM D4402 [51]. Finally, the flash and fire points of the binders were determined using the Cleveland open cup method in accordance with ASTM D92 (2018), with temperatures recorded using a calibrated thermometer [52].

#### 2.2.3. Rheological Analysis

The rheological properties of the unaged binders were evaluated to characterize their viscoelastic behavior under different temperatures and loading conditions. To assess the intermediate and high-temperature performance, dynamic shear rheometer (DSR) tests were conducted in accordance with ASTM D7175 [53]. Prior to testing, the binder samples were carefully heated under controlled conditions until a fully fluid state was achieved and then poured into silicone molds to obtain smooth and uniform surfaces. After cooling to room temperature, the specimens were trimmed to the required dimensions for testing. DSR measurements were performed using parallel plate geometry with a plate diameter of 25 mm and a gap of 1 mm. The tests were carried out under oscillatory shear loading at a constant angular frequency of 10 rad/s (1.59 Hz). Based on the applied temperature sweep, key rheological parameters, including the complex shear modulus (G*), phase angle (δ), rutting resistance parameter (G*/sinδ), and failure temperature, were determined from duplicate specimens. To evaluate the low-temperature performance of the binders, bending beam rheometer (BBR) tests were conducted comparatively on unaged binders, in accordance with ASTM D6648 [54], with two replicate specimens tested for each variable. BBR specimens were prepared by casting the molten binder into standard molds to produce prismatic beams. Prior to testing, the specimens were conditioned at −12 °C, which is a standard Superpave low-temperature specification point widely used for evaluating binder stiffness and stress relaxation behavior [54,55]. In addition, this temperature represents the minimum pavement temperature conditions of the coldest regions in Türkiye, where the present study was conducted [56]. Furthermore, several studies in the literature have adopted −12 °C as the reference temperature for BBR testing in comparative low-temperature binder evaluations [57,58]. During the test, a constant load was applied at the midspan of the beam, and the time-dependent deflection was recorded. From the measured data, the creep stiffness (S) and creep rate (m-value) at 60 s were calculated.

#### 2.2.4. Chemical Analysis

To characterize the microstructural, thermal, and chemical properties of the OSP-modified binders, scanning electron microscopy coupled with energy-dispersive X-ray spectroscopy (SEM–EDX), Fourier transform infrared (FTIR) spectroscopy, differential scanning calorimetry (DSC), and thermogravimetric analysis (TGA) were employed. These complementary techniques were selected to provide comprehensive insight into the morphology, elemental composition, functional group interactions, and thermal stability of the modified binders respectively.

The dispersion of OSP within the bitumen matrix and the associated microstructural features were examined using a scanning electron microscope equipped with an EDX detector (JEOL JSM 6510). SEM analysis was conducted to qualitatively assess the effects of OSP incorporation on surface texture, homogeneity, and film-forming behavior of the binders, as well as to identify morphological differences between the modified and reference samples. Prior to imaging, the specimens were coated with a thin gold–palladium (Au/Pd) layer to mitigate electrostatic charging and to prevent imaging artifacts such as glare, vibration, and surface distortion. SEM micrographs were used to evaluate the uniformity of OSP dispersion and interfacial characteristics within the binder matrix. Elemental presence and distribution attributable to OSP were further identified qualitatively and quantitatively through EDX analysis.

FTIR spectroscopy (PerkinElmer Spectrum 100) was utilized to investigate the chemical structure of the binders and the interactions between OSP and bitumen components. Spectra were recorded over a wavenumber range of 600–4000 cm^−1^ with a spectral resolution of 0.4 cm^−1^. This range encompasses the characteristic absorption bands of aliphatic and aromatic hydrocarbons as well as heteroatom-containing functional groups present in bitumen. FTIR analysis enabled the evaluation of changes in peak positions and intensities following modification, thereby allowing assessment of whether the modification occurred predominantly through chemical reactions or physical interactions.

DSC analyses were performed to evaluate the influence of OSP on the thermal behavior of the binders by examining phase transitions and heat flow variations. Measurements were conducted using a DSC instrument (PerkinElmer 4000) operating under a high-purity nitrogen atmosphere at a flow rate of 20 mL·min^−1^ and a heating rate of 20 °C·min^−1^. Approximately 5–10 mg of sample was placed in aluminum pans and subjected to controlled heating–cooling cycles over a temperature range of −50 to 150 °C under inert conditions.

The thermal stability and degradation behavior of the binders were determined using TGA. Analyses were carried out with a simultaneous thermal analyzer (STA 6000, PerkinElmer) under a high-purity nitrogen atmosphere at a flow rate of 20 mL·min^−1^. Samples were heated at a constant rate of 20 °C·min^−1^ over a temperature range of 30–900 °C, and the mass loss as a function of temperature was continuously recorded. The TGA results enabled comparison of the degradation behavior of bitumen and OSP components across different temperature intervals and facilitated evaluation of the effect of modification on thermal resistance.

## 3. Results

This section presents and discusses the experimental results obtained to evaluate the effects of OSP on the physical, rheological and chemical behavior of bituminous binders. The results are organized in a logical sequence, beginning with conventional physical properties, followed by rheological performance at high and low temperatures, and finally supported by microstructural and chemical analyses. Such an integrated approach enables not only the assessment of performance-related changes induced by OSP incorporation but also a deeper understanding of the underlying interaction mechanisms governing binder behavior. Where relevant, the observed trends are interpreted in relation to existing literature to highlight both consistencies and deviations.

### 3.1. Physical Properties

**Figure 6:**
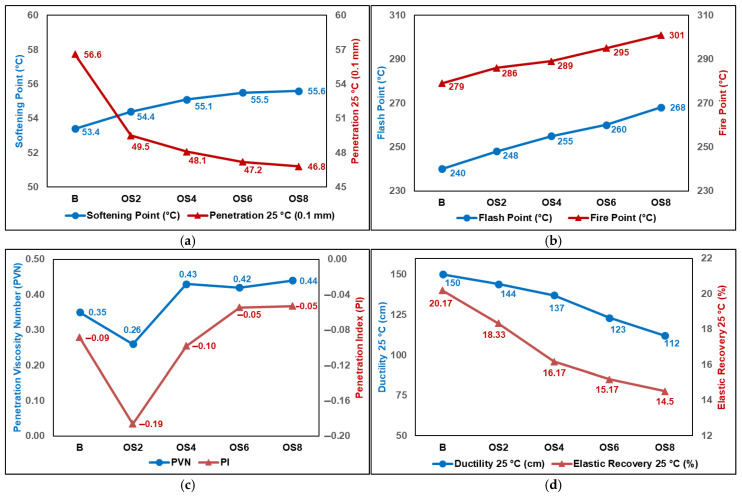

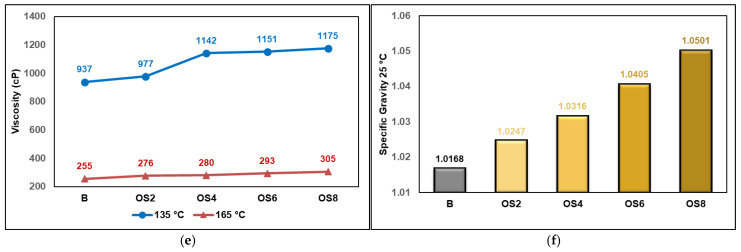
Physical test results: (**a**) softening point and penetration; (**b**) flash/fire point; (**c**) PI and PVN; (**d**) ductility and elastic recovery; (**e**) viscosity; (**f**) specific gravity.

The results of all physical tests conducted within the scope of this study are graphically presented in Figure 6. As shown in Figure 6a, although a pronounced reduction in penetration was observed for the binder modified with 2% OSP, the penetration values decreased more gradually and uniformly with increasing OSP content. Compared with the neat binder, the penetration values of OS2, OS4, OS6, and OS8 binders were reduced by 12.5%, 15.0%, 16.6%, and 17.3%, respectively. From a practical standpoint, this controlled reduction in penetration indicates a moderate increase in binder stiffness, which is generally associated with improved resistance to permanent deformation at elevated service temperatures. Importantly, the magnitude of penetration reduction remains within ranges that do not indicate excessive hardening, suggesting that binder workability and constructability are not adversely affected. The softening point results exhibited a trend consistent with the penetration data, showing a gradual increase with increasing OSP content. Even for the OS8 binder, which exhibited the highest increase, the softening point rose by only 4.1%. This moderate increase implies enhanced resistance to softening under high-temperature conditions without a drastic shift in binder consistency. The combined trends of decreasing penetration and increasing softening point suggest a reduction in temperature susceptibility rather than an abrupt stiffening effect. These observations are consistent with findings reported for binders modified with olive husk ash (OHA) and olive kernel ash (OKA) in previous studies [28,29].

The flash and fire point results shown in Figure 6b reveal that the incorporation of OSP led to a nearly linear increase in both parameters, indicating enhanced resistance of the binders to ignition and combustion. As the OSP content increased, the safe operating temperature range of the binders at elevated temperatures was effectively extended. The temperature susceptibility of the binders was evaluated using the Penetration Index (PI) and the Penetration Viscosity Number (PVN), calculated according to Equations (1) and (2).

(1) $$PI=\frac{\left(1952-500*\log\left({Pen}_{\left(25\;{}^{\circ}C\right)}\right)-20*SP\right)}{\left(50*\log\left({Pen}_{\left(25\;{}^{\circ}C\right)}\right)-SP-120\right)}$$

(2) $$PVN=-1.5*\left(\frac{4.258-0.7967*\log\left({Pen}_{\left(25\;{}^{\circ}C\right)}\right)-\log\left(V_{\left(135\;{}^{\circ}C\right)}\right)}{0.795-0.1858*\log\left({Pen}_{\left(25\;{}^{\circ}C\right)}\right)}\right)$$

where

${Pen}_{\left(25\;{}^{\circ}C\right)}$ is the penetration value measured at 25 °C (0.1 mm);
$V_{\left(135\;{}^{\circ}C\right)}$ is the rotational viscosity measured at 135 °C (Pa·s);
SP is the softening point temperature (°C).

PI and PVN are widely used empirical indicators reflecting the binder’s sensitivity to temperature variations and the balance between penetration and viscosity, respectively. It should be noted that both indices are calculated from experimentally measured parameters and therefore inherently include the cumulative uncertainty associated with penetration, viscosity, and softening point tests. Consequently, PI and PVN values are more appropriately interpreted in a relative and comparative manner, with emphasis on trends rather than absolute magnitudes. Within this context, the calculated PI values ranged from −0.19 to −0.05, while the PVN values varied between 0.26 and 0.44. The fact that all modified binders exhibited PI values within the commonly accepted range of −1 to +1 indicates that the incorporation of OSP maintained the temperature susceptibility of the binders within acceptable limits despite minor variations. As illustrated in Figure 6c, the OS2 binder exhibited the lowest PI (−0.19) and PVN (0.26) values, suggesting that low OSP contents may slightly increase the binder’s sensitivity to temperature variations. With increasing OSP dosage, the PI values gradually increased toward approximately −0.05 and the PVN values stabilized within a narrow range of 0.42–0.44. Considering the empirical nature and inherent uncertainty of these indices, this consistent stabilization trend indicates that, particularly for OS4, OS6, and OS8 binders, OSP contributes to a more balanced penetration–viscosity relationship and improved thermal stability without introducing excessive variability.

As illustrated in Figure 6d, ductility values decreased with increasing OSP content, as expected, due to the reduction in tensile deformation capacity caused by the presence of OSP particles. This behavior suggests that OSP slightly embrittles the neat bitumen, thereby reducing its tensile strain capacity. The reductions in ductility became more pronounced at OSP contents of 6% and 8%, reaching decreases of 18.0% and 25.3%, respectively, compared with the neat binder. A similar trend was observed for elastic recovery, which closely followed the ductility results. For the OS8 binder, where the maximum reduction was recorded, elastic recovery decreased by 28.1% relative to the control binder. The results of both the elastic recovery and ductility tests indicate that the modified binders exhibit similar trends in terms of their elastic and plastic deformation capacities. Moreover, the ductility values obtained for all samples remained above the minimum limit (100 cm) specified by the relevant standard [42]. Figure 6e shows that the addition of OSP resulted in an increase in binder viscosity at both test temperatures. RVT measurements showed moderate increases in viscosity for OS8, reaching 25.4% at 135 °C and 19.6% at 165 °C, without indicating excessive binder stiffening. This increase followed a gradual and consistent trend with rising OSP content, demonstrating that OSP enhances the flow resistance of the binder. Nevertheless, the viscosity values of all modified binders at 135 °C remained below the commonly accepted upper limit of 3000 cP for hot mix asphalt production [55], indicating that OSP does not excessively impair workability. These viscosity results suggest that OSP has the potential to improve the high-temperature performance of bitumen and may contribute positively to rutting resistance. Moreover, the relatively limited change in viscosity with increasing temperature indicates that the incorporation of OSP does not excessively increase the temperature susceptibility of the binder. Figure 6f presents the specific gravity results obtained using a pycnometer. Prior to modification, the specific gravity of OSP was measured using the same method and determined to be 1.5055. Since the specific gravity of OSP is higher than that of the neat bitumen, the incorporation of OSP resulted in a slight increase in the specific gravity of the modified binders.

When the physical test results are considered collectively, the incorporation of OSP is found to induce a balanced modification in the fundamental consistency and workability characteristics of the bituminous binder without causing adverse effects. Overall, the physical test findings suggest that OSP can be regarded as an environmentally friendly biopolymer capable of improving high-temperature performance while preserving acceptable consistency and handling characteristics of bituminous binders.

### 3.2. Rheological Performance

#### 3.2.1. DSR Results

Rutting is a critical form of permanent deformation in asphalt pavements, mainly occurring under high temperatures and repeated heavy traffic loading. It is primarily associated with insufficient binder stiffness and a dominant viscous response at elevated temperatures [59,60]. Therefore, accurate evaluation of binder viscoelastic properties using the dynamic shear rheometer (DSR) is essential for assessing high-temperature pavement performance. The complex shear modulus (G*), phase angle (δ), and the rutting factor (G*/sinδ) derived from these parameters are the principal rheological indicators used to characterize the viscoelastic behavior of asphalt binders at intermediate and high temperatures. The G* value represents the total resistance of the binder to deformation under oscillatory shear loading, whereas the phase angle (δ) reflects the balance between elastic and viscous components of the binder response. Lower δ values indicate more elastic behavior, while higher values signify a more viscous response. Joint evaluation of these parameters is therefore critical for understanding how a binder responds mechanically under service temperature conditions. Another important parameter derived from DSR results is the failure temperature, defined as the highest temperature at which the unaged binder satisfies the rutting criterion of G/sinδ ≥ 1.0 kPa. For RTFO-aged binders, this parameter is determined based on the temperature corresponding to the rutting criterion of G/sinδ ≥ 2.2 kPa. DSR testing on unaged binders is particularly valuable for assessing the intrinsic high-temperature behavior imparted by modifiers under initial service conditions and for isolating the direct effects of modification [61].

**Figure 7:**
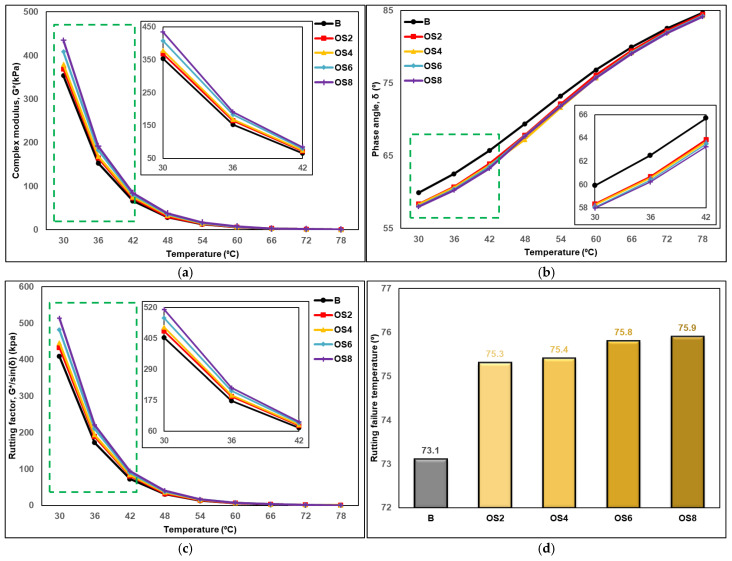
DSR test results for unaged binders: (**a**) complex modulus; (**b**) phase angle; (**c**) rutting factor; (**d**) rutting failure temperature. The dashed green boxes indicate the regions enlarged in the inset zoomed-in graphs.

As shown in the curves presented in Figure 7a,b, an increase in temperature resulted in a decrease in G* and a corresponding increase in δ for all unaged binders. This trend is consistent with the viscoelastic nature of bitumen, indicating a progressive shift toward more viscous behavior as temperature rises. Nevertheless, the OSP-modified binders exhibited higher G* values and lower or comparable δ values across the entire temperature range when compared with the neat binder. In particular, as illustrated in Figure 7c, the rutting factor (G*/sinδ) clearly demonstrates that the incorporation of OSP enhances the resistance of the binder to deformation at elevated temperatures. The G*/sinδ values increased monotonically with increasing OSP content at all temperatures, with the most pronounced improvement observed in the 30–42 °C range. This temperature interval corresponds to the intermediate-to-high service temperatures typically considered in binder-level rheological evaluations, and the results indicate that OSP contributes positively to high-temperature binder performance in terms of increased resistance to permanent deformation. These findings provide valuable insight into the potential role of OSP in influencing rutting-related mechanisms at the binder scale and may inform future mixture-level investigations.

Examination of the phase angle results in Figure 7b reveals that OSP incorporation did not shift the viscoelastic balance entirely toward viscous behavior. On the contrary, the modified binders exhibited phase angle values comparable to or slightly lower (by approximately 2°) than those of the neat binder. When considered alongside the increased G*/sinδ values, this behavior suggests that OSP acts as a physical modifier that enhances binder stiffness without inducing excessive hardening. In other words, OSP improves resistance to flow at high temperatures while preserving the elastic component of the binder response. The failure temperatures shown in Figure 7d further confirm the beneficial effect of OSP on high-temperature performance. The failure temperatures of the OSP-modified binders exhibited a gradual and systematic increase relative to the neat binder, with the magnitude of improvement becoming more pronounced as the OSP content increased. Compared to the neat binder, the 2.8 °C (almost 3) increase observed for the OS8-modified binder indicates a measurable improvement in high-temperature performance. The observed increase in failure temperature is consistent with the monotonic rise in G*/sinδ values and confirms that OSP enhances resistance to high-temperature deformation within the binder matrix. Importantly, the absence of abrupt or excessive increases in failure temperature indicates that OSP improves high-temperature performance in a controlled manner without excessively stiffening the binder. This finding suggests that OSP can be regarded as a modifier that enhances the high-temperature performance of unaged binders while maintaining workability and a balanced viscoelastic response.

#### 3.2.2. BBR Results

Thermal cracking in asphalt pavements at low temperatures is mainly associated with thermal contraction and insufficient stress relaxation of the binder. Accordingly, the bending beam rheometer (BBR) test is commonly used to characterize low-temperature binder behavior through creep stiffness (S) and m-value parameters. The combined S/m-value provides a concise indicator of the balance between stiffness and stress relaxation, where higher values indicate stiffness-dominated behavior and increased thermal cracking susceptibility, while lower values reflect a more favorable low-temperature response [62,63,64].

**Figure 8:**
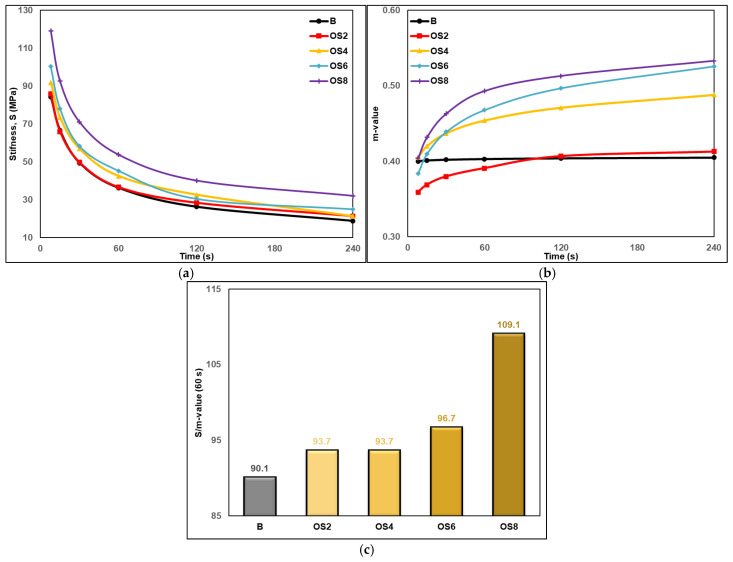
BBR test results for unaged binders: (**a**) creep stiffness; (**b**) m-value; (**c**) S/m-value.

As shown in Figure 8a, the creep stiffness (S) values obtained from the BBR test exhibited a decreasing trend with time for all unaged binders, reflecting the time-dependent stress relaxation behavior at low temperatures. However, the OSP-modified binders consistently exhibited higher S values than the neat binder over the entire loading duration. This trend indicates that the incorporation of OSP increases binder stiffness at low temperatures, with the effect becoming more pronounced as the OSP content increases. In particular, the OS8 binder displayed the highest stiffness values throughout the entire measurement period, demonstrating a strong correlation between OSP content and increased low-temperature stiffness.

As illustrated in Figure 8b, the curves clearly indicate that OSP exerts a pronounced influence on the stress relaxation behavior of the binders at low temperatures. While the neat binder exhibited an approximately constant m-value, the m-values of the OSP-modified binders increased with time. In particular, the OS4, OS6, and OS8 binders reached higher m-values at 60 s compared with the neat binder, indicating a more effective time-dependent stress relaxation at low temperatures. This trend suggests that, up to certain dosages, the incorporation of OSP not only increases binder stiffness but also promotes viscoelastic relaxation mechanisms that facilitate stress dissipation over time. However, the m-value responses varied depending on the OSP content. For the OS2 binder with a relatively low OSP dosage, the increase in m-value remained limited, whereas a pronounced enhancement in m-values was observed at moderate to high dosages (OS4, OS6, and OS8). This behavior implies that OSP establishes more effective microstructural interactions within the binder matrix beyond a certain threshold content, thereby enhancing stress relaxation capacity at low temperatures. The S/m-value results presented in Figure 8c provide a more comprehensive assessment of low-temperature performance. Although the neat binder exhibited the lowest S/m-value, a systematic increase in this ratio was observed with increasing OSP content. The OS2 and OS4 binders showed only a limited increase in S/m-value relative to the neat binder, indicating a comparatively balanced low-temperature response, where the increase in stiffness was partially offset by adequate stress relaxation capacity. In contrast, the OS6 and especially the OS8 binders exhibited noticeably higher S/m-values, indicating that stiffness enhancement became dominant over stress relaxation, thereby approaching the upper practical limits for low-temperature performance and suggesting an increased susceptibility to thermal cracking. Overall, the results indicate that the effect of OSP on low-temperature performance is highly dosage-dependent. Moderate OSP contents enhance the stress relaxation capability of the binder while limiting stiffness increase to acceptable levels; however, higher OSP contents clearly lead to stiffness-dominated rheological behavior, as evidenced by elevated S and S/m-values, thereby increasing the potential for thermal cracking. When considered together with the DSR results, these findings highlight that although OSP effectively improves high-temperature performance, identifying an optimal dosage is critical to achieving a balanced improvement in both high- and low-temperature behavior of bituminous binders.

### 3.3. Chemical Characterization

#### 3.3.1. SEM-EDX Analysis

**Figure 9:**
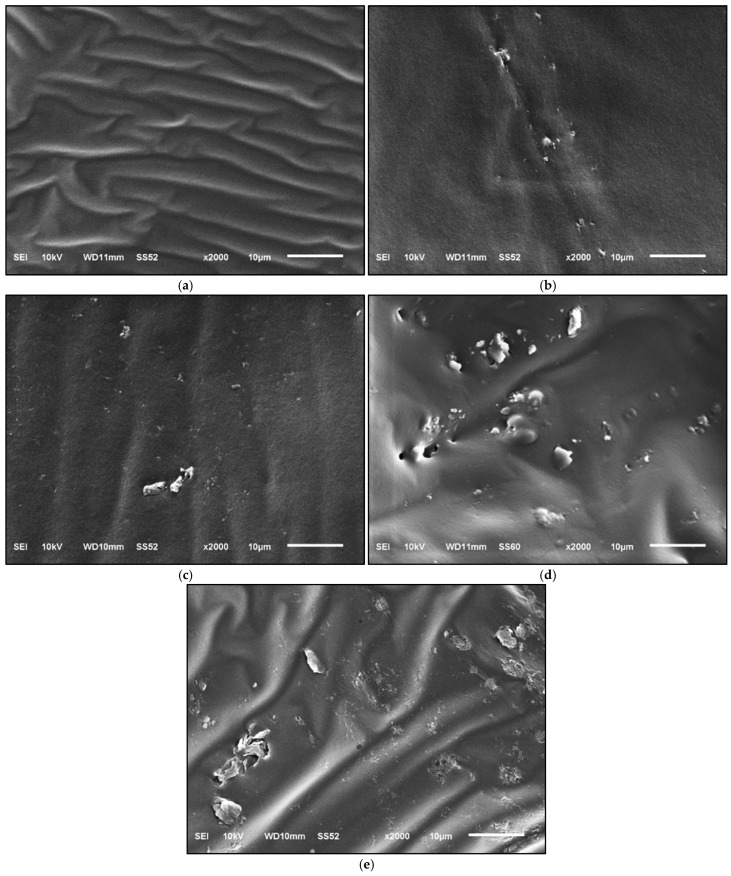
SEM images: (**a**) B 50/70; (**b**) OS2; (**c**) OS4; (**d**) OS6; (**e**) OS8.

The SEM micrographs presented in Figure 9 clearly illustrate the influence of OSP incorporation on the microstructure of the bituminous binder. As observed in Figure 9a, the SEM image of the neat binder exhibits a continuous, wavy, and homogeneous surface morphology, which is characteristic of the fluid and viscoelastic nature of bitumen. In the SEM image of the OS2 binder shown in Figure 9b, corresponding to a low OSP dosage, small dispersed particles are observed within the binder matrix, most of which appear to be well embedded and encapsulated by the surrounding bitumen. As the OSP content increases to moderate and high levels in the OS4, OS6, and OS8 binders (Figure 9c–e), a noticeable increase in both the number and surface visibility of OSP particles is observed. These images indicate the formation of locally concentrated regions of OSP particles within the bitumen matrix; however, the overall continuity of the binder matrix remains largely preserved. This morphological configuration suggests that OSP acts in a filler-like manner within the binder while partially restricting binder flow, thereby contributing to an increase in stiffness. Overall, the SEM observations indicate that OSP modifies the binder predominantly through physical and microstructural interactions rather than through chemical reactions. It was determined that OSP exhibited a homogeneous dispersion within the bitumen matrix at all dosage levels, as also confirmed by the smear test conducted during mixture preparation; this structure preserved the stiffness–viscoelasticity balance of the binder while leading to a gradual increase in rigidity. SEM observations show that at low OSP contents, particles are homogeneously dispersed within the binder matrix, whereas higher dosages lead to localized agglomeration. This microstructural evolution explains the observed rheological behavior, where improved dispersion enhances interfacial interactions and results in increased viscosity and complex modulus (G*). At higher OSP contents, particle clustering may introduce heterogeneity and stress concentrations, which can limit further improvements in rutting-related performance despite continued viscosity increase. In addition, DSC results indicate that OSP affects the mobility of the binder’s amorphous phase, which is commonly associated with low-temperature stiffness. Overall, the combined SEM and DSC findings suggest a dosage-dependent, predominantly physical modification mechanism consistent with the measured rheological response.

The EDX analysis results of binders modified with different OSP contents reveal limited yet meaningful changes in elemental composition as a function of OSP dosage. Carbon (C), oxygen (O), and sulfur (S) were identified as the dominant elements in both the neat binder and all OSP-modified binders, which is consistent with the hydrocarbon-based nature of bitumen and the lignocellulosic origin of OSP. The relatively increased oxygen content observed with OSP incorporation suggests the introduction of oxygen-containing functional groups associated with phenolic compounds, lignin, and cellulose present in the OSP structure. Notably, trace elements such as phosphorus (P), calcium (Ca), and zinc (Zn), as reported in Table 4, exhibited gradual or limited increases with rising OSP content. Previous studies have shown that OSP and similar biomass-derived wastes are naturally rich in phenolic acids, tocopherols, and phosphorus-containing organic compounds, and that elements such as P and Zn may coexist with antioxidant-related constituents in biomass-based materials [65]. Overall, the EDX results indicate that the incorporation of OSP introduces biomass-derived elements associated with antioxidant functionality into the binder matrix without causing excessive or abrupt changes in elemental composition.

#### 3.3.2. FTIR Analysis

**Figure 10:**
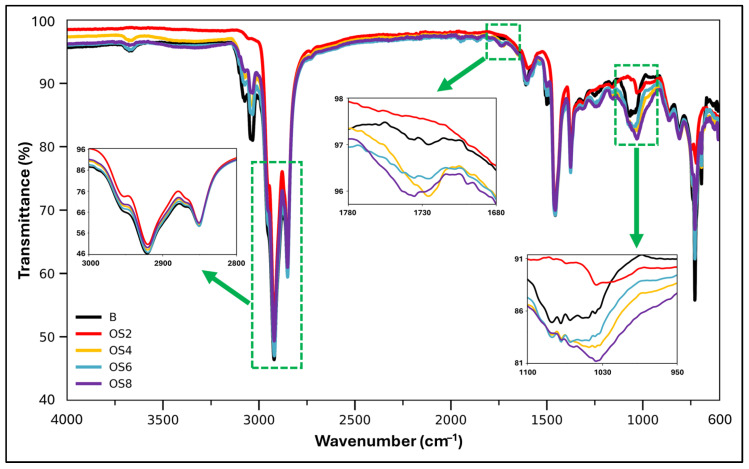
FTIR spectrum.

The FTIR spectra of the neat binder and binders modified with different OSP contents were comparatively analyzed over the wavenumber range of 600–4000 cm^−1^. The preservation of the characteristic absorption bands of bitumen in all spectra indicates that the incorporation of OSP did not induce the formation of new chemical bonds or functional groups within the binder structure. This observation suggests that OSP influences the binder predominantly through physical and microstructural interactions rather than through chemical reactions. As shown in Figure 10, the broad band centered around 3432 cm^−1^ in the high-wavenumber region is attributed to O–H stretching vibrations, indicating the presence of hydrogen-bonded hydroxyl groups. This band is weak in the neat binder but becomes increasingly pronounced with rising OSP content, particularly in the OS6 and OS8 binders. The absorption band observed at 3032 cm^−1^ corresponds to aromatic =C–H stretching vibrations. The consistent position and character of this band across all samples demonstrate that OSP incorporation does not disrupt the aromatic core structure of the bitumen. Similarly, the dominant bands at 2921 cm^−1^ and 2852 cm^−1^, associated with the asymmetric and symmetric stretching vibrations of aliphatic –CH_2_ groups, respectively, are clearly present in all spectra. With increasing OSP content, limited yet systematic variations in the intensities of these bands were observed, indicating that the interaction between bitumen and OSP affects the local molecular environment of the aliphatic chains. However, the absence of peak shifts confirms that no chemical bond cleavage or formation occurred [65,66]. In the mid-wavenumber region, the band at approximately 1731 cm^−1^ is attributed to C=O stretching vibrations associated with ester or acidic carbonyl groups. No new peaks emerged in this region following OSP addition; nevertheless, noticeable changes in band shape and intensity were observed for the OS6 and OS8 binders. These variations suggest that oxygen-containing components derived from OSP interact with aromatic and carbonyl structures in the binder through environmental vibrational interactions rather than through chemical oxidation reactions. The band at 1604 cm^−1^, corresponding to aromatic C=C stretching vibrations, remained intact in all samples, confirming that the structural integrity of the aromatic fraction of bitumen was preserved after modification [66].

At lower wavenumbers, the bands at 1457 cm^−1^ and 1376 cm^−1^ are attributed to the bending vibrations of –CH_2_ and –CH_3_ groups, respectively, reflecting the stability of the aliphatic backbone. The consistent presence of these bands across all binders further confirms that OSP incorporation does not alter the fundamental hydrocarbon structure of bitumen. In contrast, the band observed at approximately 1031 cm^−1^, associated with C–O stretching vibrations, becomes more pronounced with increasing OSP content. This band can be regarded as one of the most distinctive FTIR signatures of oxygen-containing functional groups originating from the lignocellulosic structure of OSP. Additionally, the aromatic C–H out-of-plane bending vibration observed at 873 cm^−1^ was preserved in all samples, indicating the continued presence of aromatic structures after modification [65,66]. Overall, the FTIR results demonstrate that OSP incorporation does not lead to the formation of new chemical functional groups in the bituminous binder. Instead, the intensity and shape variations observed in oxygen-related bands, particularly at 3432, 1731, and 1031 cm^−1^, indicate that OSP exerts its influence through physical and microstructural interactions within the binder matrix. When evaluated together with the presence of elements such as oxygen, phosphorus, and zinc reported in the EDX analysis and the particle–matrix interactions observed in SEM images, these findings suggest that OSP provides a dosage-dependent, non-reactive modification mechanism with potential antioxidant functionality. This interpretation of the FTIR results is also consistent with the rheological performance trends observed in the DSR and BBR tests.

#### 3.3.3. DSC Analysis

**Figure 11:**
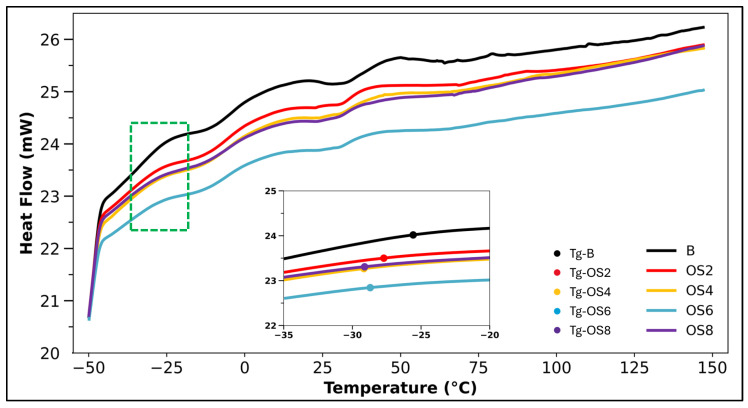
DSC results. The dashed green box highlights the inset zoom corresponding to the glass transition region.

DSC analysis was conducted to investigate the thermal behavior of the neat and OSP-modified binders, and the resulting heat flow–temperature curves are presented in Figure 9. Owing to the broad temperature range over which the glass transition occurs in bituminous binders, the glass transition temperature (Tg) was determined based on the region of maximum slope on the heat flow curves, within the temperature interval of −35 to −20 °C, as illustrated in Figure 11. This region is representative of molecular mobility in bitumen at low temperatures. The determined Tg values are summarized in Table 5. The neat binder exhibited a Tg of approximately −25.58 °C, while the incorporation of OSP led to a progressive decrease in Tg, reaching −27.71 °C and −29.16 °C for the OS2 and OS4 binders, respectively. This reduction in Tg indicates that OSP increases the free volume within the bitumen matrix, thereby enhancing molecular chain mobility and promoting a more flexible structure at low temperatures. Although the addition of OSP resulted in a noticeable decrease in Tg, the enthalpy change associated with the glass transition (ΔH) remained at comparable levels for all samples. This observation suggests that while OSP enhances molecular mobility, it does not disrupt the fundamental amorphous structure or compromise the thermal integrity of the binder. The ΔH values were obtained by integrating the heat flow signal over the glass transition temperature interval in the DSC curves.

At intermediate and high temperature ranges, the absence of sharp endothermic or exothermic peaks in the DSC curves indicates that OSP incorporation does not induce phase separation or unstable thermal transitions within the bitumen. Moreover, this behavior confirms that the addition of OSP does not adversely affect the high-temperature thermal stability of the binder. These findings are consistent with the homogeneous dispersion observed in SEM analyses and the absence of new chemical bond formation detected in FTIR results, collectively confirming that OSP interacts with bitumen predominantly through physical mechanisms. Overall, the DSC results demonstrate that OSP modification provides a balanced effect by improving low-temperature performance while preserving high-temperature thermal behavior. When evaluated together with the FTIR and SEM findings, the results indicate that OSP can be considered a thermally compatible, sustainable biopolymer that enhances low-temperature flexibility in bituminous binders without compromising their thermal stability.

#### 3.3.4. TGA

**Figure 12:**
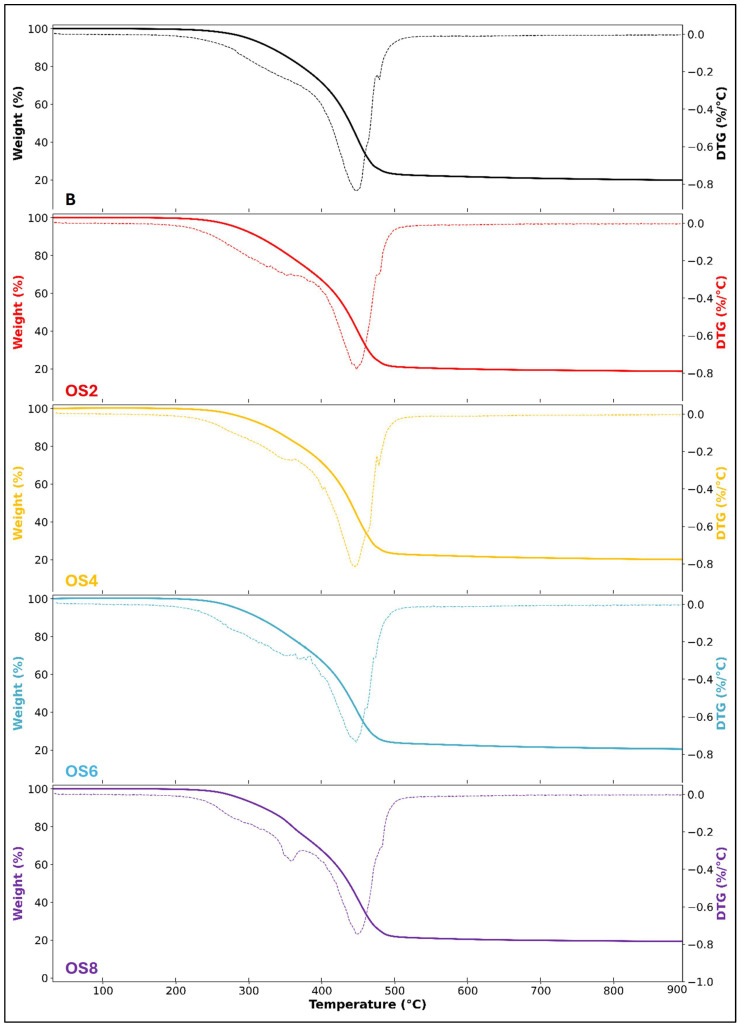
TGA curves. The solid colored lines represent the thermogravimetric curves, while the dashed colored lines correspond to the DTG curves.

TGA was conducted to evaluate thermal stability, thermal degradation behavior, and the effects of OSP modification on the binders. The TGA results are presented in Figure 12. The thermogravimetric curves indicate that the incorporation of OSP led to controlled and systematic changes in the thermal degradation behavior of the binders compared with the neat binder. The onset thermal degradation temperatures (OTDT), determined using the tangent method from the TGA curves and summarized in Table 6, were found to range between 371.09 and 374.67 °C, exhibiting only minor variations among the samples. Examination of the derivative thermogravimetry (DTG) curves revealed that the peak temperatures corresponding to the maximum degradation rate were concentrated within a narrow range of 449.09–451.31 °C for all binders. The DTG peak temperature of the neat binder, determined as 450.90 °C, did not exhibit a significant shift following OSP incorporation. Similarly, no pronounced differences were observed in the residual mass values measured in the high-temperature region. These results indicate that OSP does not alter the fundamental thermal degradation mechanism of bitumen. Instead, it plays a regulatory role in the degradation of kinetics. In other words, while the primary decomposition characteristics of the hydrocarbon backbone are preserved, the presence of OSP moderates the distribution and rate of the degradation process. Overall, the TGA findings demonstrate that OSP functions as a supportive modifier that maintains the intrinsic thermal degradation behavior of bitumen while contributing to improved thermal stability and high-temperature performance. This behavior further confirms that OSP can be effectively incorporated into bituminous binders without compromising their inherent thermal resistance characteristics.

## 4. Conclusions

In this study, the feasibility of using OSP as a sustainable biopolymer for bituminous binders was comprehensively evaluated through an integrated experimental approach encompassing physical, rheological, and chemical analyses. The primary motivation of this study is to convert OS, an agricultural by-product, into a high-value pavement material, thereby reducing environmental impacts and improving the performance of bituminous binders. To this end, neat and OSP-modified binders at different dosages were prepared and systematically examined using a multi-scale experimental methodology. Based on the experimental findings, the main conclusions of this study can be summarized as follows:

- Conventional physical tests showed that OSP incorporation leads to generally linear changes in binder properties. At the highest dosage (OS8), penetration decreased by 17.3% and the softening point increased by 4.1%, indicating improved resistance to temperature-induced softening. PI (−0.19 to −0.05) and PVN (0.26–0.44) values remained within acceptable ranges, while rotational viscosity results confirmed that all binders maintained adequate workability despite moderate viscosity increases at high OSP contents.
- Rheological evaluations demonstrated a strong dosage-dependent behavior. DSR results revealed a progressive increase in failure temperature, reaching an improvement of approximately 2.8 °C for OS8, indicating enhanced high-temperature rutting resistance. In contrast, BBR results showed that S/m-values increased only moderately up to OS6 (~7.3%) but rose sharply for OS8 (~20%), reflecting stiffness-dominated behavior at high OSP contents.
- Optimal performance balance was achieved at moderate OSP dosages (OS4–OS6), which significantly improved high-temperature performance while avoiding excessive stiffness and adverse low-temperature effects under the applied laboratory conditions.
- Microstructural and thermal analyses confirmed that OSP modification is predominantly physical in nature. FTIR analysis showed no formation of new functional groups, SEM–EDX observations indicated a generally homogeneous dispersion of OSP particles, and DSC/TGA results demonstrated preserved thermal stability with a moderate reduction in glass transition temperature.
- Sustainability considerations highlight that the use of OSP, an agricultural by-product, provides an environmentally and economically viable alternative to conventional modifiers by valorizing waste materials in line with circular economic principles.
- Overall, the findings confirm that the performance of OSP-modified binders is highly dosage dependent; excessive OSP contents may increase brittleness and hinder workability, whereas careful optimization of OSP content enables balanced improvements in binder performance.

In conclusion, within the scope of this study and based on the applied laboratory-scale physical, rheological, and thermal indicators, OSP can be regarded as an environmentally friendly, biopolymer-based modifier that enhances selected performance-related properties of bituminous binders at appropriate dosages. Future research should focus on investigating the short- and long-term aging behavior of OSP-modified binders, conducting mechanical and performance-based tests at the asphalt mixture level, and evaluating field applications to facilitate the transfer of laboratory findings into engineering practice.

## Figures and Tables

**Table 1:** Summary of previous studies on olive-derived materials.

Reference	Material	Process	Application	Improvements	Limitations
[27,28]	ash-based; OKA	incinerated at 600 °C	binder & mixture	improved rutting and fatigue resistance increased shear modulus & recovery enhanced elastic response	decreased fatigue life reduced low-temperature performance decreased workability
[29,30,31]	ash-based; OHA	incinerated at 400 °C	binder & mixture	improved Marshall stability & stiffness higher thermal resistance reduced permanent deformation	no rheological analysis decreased flow & flexibility limited temperature range
[32]	raw; OP	dried & grounded	binder & mixture	improved rutting & fatigue resistance lower thermal susceptibility cost-effective SBS alternative	decreased workability, ductility & poor storage stability at high dosages
[33]	extracted; OPL	sulfuric acid hydrolysis	binder & mixture	improved thermal stability, rutting & aging resistance cost savings vs polymer modifiers	phase separation & low ductility at high dosage dosage-sensitive behavior
[34]	processed; OP	dried & grounded	binder	improved binder–aggregate adhesion stable antioxidant activity after heating suitable for preservation treatments	moisture-sensitive processing decreased workability at high dosage
[35]	processed; OP	dried & grounded	binder & mixture	retarded oxidative hardening improved cracking & fatigue resistance	decreased workability at high dosage limited effect on rutting
[36]	ash-based; OWA	incinerated at 600 °C	binder & mixture	increased Marshall stability (≤10%) improved rutting resistance enhanced retained stability	increased air voids reduced dynamic modulus lost stability beyond optimum
[37]	processed; OP	incinerated at 300–350 °C	binder & mixture	increased moisture resistance improved Superpave mix performance	limited high-temperature performance no rheological analysis
[38]	extracted; OPO	extracted at 120 °C	binder	balanced performance (all temperatures) upgraded PG at both high & low ends improved rutting & cracking resistance	higher material cost than bio-oil alone multi-step modification process
[39]	processed; OLR	grounded	binder	improved oxidative aging resistance rheological enhancement after aging	dosage sensitivity no rutting-focused performance tests
[40]	raw; OMW	washed & sieved	mixture	enhanced skid resistance improved surface micro texture	high replacement may affect strength limited binder rheology analysis
[41]	processed; OP	dried & grounded	binder	improved stripping resistance enhanced fatigue/cracking resistance better aggregate–binder adhesion	reduced High-temperature stiffness only conventional tests softening effect may increase rutting

Abbreviations: OKA, olive kernel ash; OHA, olive husk ash; OP, olive pomace; OPL, olive pomace lignin; OWA, olive waste ash; OPO, olive pomace oil; OLR, olive leaf residue; OMW, olive mill waste; PG, performance grade.

**Table 2:** Characteristics of B 50/70 bitumen.

Characteristic	Value	Criteria (EN 12591)
Penetration at 25 °C (0.1 mm)	59.5	50–70
Softening Point (°C)	53.1	46–54
Flash Point (°C)	240	≥230
Ductility at 25 °C (cm)	150	≥100
Specific Gravity at 25 °C	1.0155	1.01–1.06
Mass Loss (%)	0.24	≤0.50
Penetration Index (PI)	−0.03	-

**Table 3:** EDX Analysis of OSP.

Element	C	O	Zn	N	Si	Fe	K	Ca	Al	Ni	Mn	Mg	Total
wt. (%)	58.559	34.83	2.442	1.397	1.023	0.421	0.401	0.295	0.210	0.153	0.138	0.132	100

**Table 4:** EDX analysis results.

Element (%)	B	OS2	OS4	OS6	OS8
C	76.632	76.173	76.757	76.814	76.387
O	14.378	14.521	13.473	13.348	13.692
Mg	0.053	0.040	0.076	0.015	0.049
P	0.240	0.236	0.273	0.344	0.344
S	8.483	8.730	9.129	9.050	8.989
Ca	0.044	0.104	0.069	0.168	0.185
Zn	0.170	0.196	0.223	0.261	0.354
Total	100.000	100.000	100.000	100.000	100.000

**Table 5:** DSC-derived glass transition parameters.

Sample	Tg (°C)	Tg Onset (°C)	Tg End (°C)	ΔH (J/g)
B	−25.58	−39.96	−13.08	~2.27
OS2	−27.71	−41.27	−14.55	~2.15
OS4	−29.16	−43.19	−10.07	~2.19
OS6	−28.70	−42.81	−13.21	~2.18
OS8	−29.11	−42.70	−14.28	~2.23

**Table 6:** Thermal characteristics of bitumen samples with TGA.

Sample	OTDT (°C)	DTG Peak Value (°C)	Residue Mass (%)
B	374.67	450.90	19.87
OS2	371.09	449.09	18.71
OS4	372.65	450.11	20.09
OS6	373.77	450.45	20.56
OS8	373.34	451.31	19.30

## Data Availability

The original contributions presented in this study are included in the article.
